# A Study on the Correlation between the Oxidation Degree of Oxidized Sodium Alginate on Its Degradability and Gelation

**DOI:** 10.3390/polym14091679

**Published:** 2022-04-21

**Authors:** Hongcai Wang, Xiuqiong Chen, Yanshi Wen, Dongze Li, Xiuying Sun, Zhaowen Liu, Huiqiong Yan, Qiang Lin

**Affiliations:** 1 Key Laboratory of Tropical Medicinal Resource Chemistry of Ministry of Education, College of Chemistry and Chemical Engineering, Hainan Normal University, Haikou 571158, China; whcz0505@163.com (H.W.); wenyanshi2022@163.com (Y.W.); 2 Key Laboratory of Natural Polymer Functional Material of Haikou City, College of Chemistry and Chemical Engineering, Hainan Normal University, Haikou 571158, China; chenxiuqiongedu@163.com (X.C.); dongzeli2019@163.com (D.L.); sunxiuying2019@163.com (X.S.); liuzhaowenyifan@126.com (Z.L.); 3 Key Laboratory of Water Pollution Treatment & Resource Reuse of Hainan Province, College of Chemistry and Chemical Engineering, Hainan Normal University, Haikou 571158, China; 4 College of Pharmacy, Gannan Medical University, Ganzhou 341000, China

**Keywords:** oxidized sodium alginate, oxidation degree, biodegradation gelation ability, rheological properties

## Abstract

Oxidized sodium alginate (OSA) is selected as an appropriate material to be extensively applied in regenerative medicine, 3D-printed/composite scaffolds, and tissue engineering for its excellent physicochemical properties and biodegradability. However, few literatures have systematically investigated the structure and properties of the resultant OSA and the effect of the oxidation degree (OD) of alginate on its biodegradability and gelation ability. Herein, we used NaIO_4_ as the oxidant to oxidize adjacent hydroxyl groups at the C-2 and C-3 positions on alginate uronic acid monomer to obtain OSA with various ODs. The structure and physicochemical properties of OSA were evaluated by Fourier transform infrared spectroscopy (FT-IR), ^1^H nuclear magnetic resonance (^1^H NMR), X-ray Photoelectron Spectroscopy (XPS), X-ray Diffraction (XRD), and thermogravimetric analysis (TGA). At the same time, gel permeation chromatography (GPC) and a rheometer were used to determine the hydrogel-forming ability and biodegradation performance of OSA. The results showed that the two adjacent hydroxyl groups of alginate uronic acid units were successfully oxidized to form the aldehyde groups; as the amount of NaIO_4_ increased, the OD of OSA gradually increased, the molecular weight decreased, the gelation ability continued to weaken, and degradation performance obviously rose. It is shown that OSA with various ODs could be prepared by regulating the molar ratio of NaIO_4_ and sodium alginate (SA), which could greatly broaden the application of OSA-based hydrogel in tissue engineering, controlled drug release, 3D printing, and the biomedical field.

## 1. Introduction

**Figure 1:**
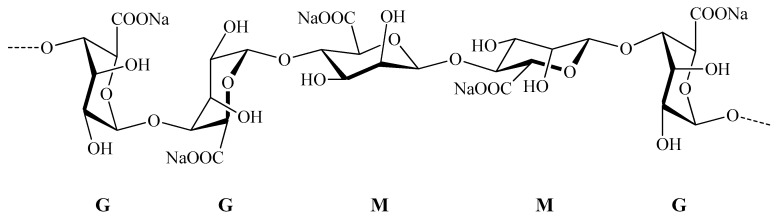
Molecular structure of sodium alginate.

Sodium alginate (SA), an irregular linear natural polysaccharide polymer derived from algae and bacteria, comprises 1,4-linked β-d-mannuronic acid (M Block) and 1,4-linked α-l-guluronic acid (G Block) with a homogeneous (poly-G, poly-M) or heterogeneous (MG) block composition [1,2], as illustrated in Figure 1. Owing to the advantages of good biocompatibility, low toxicity, non-immunogenicity, reproducibility, and plasticity, alginate is widely used in wound dressing, tissue engineering, pharmaceutical industries, food, and cosmetics [3,4,5,6]. However, as a result of the numerous carboxyl and hydroxyl groups on its molecular backbone, the hydrophilic alginate suffers from uncontrollable degradation and massive swelling properties, leading to its weak stability in biological buffers, which significantly restricts its practical application in the biomedical field [7,8]. In addition, SA with high-molecular-weight alginate hardly degrades in the body because of the lack of alginate-degrading enzymes [9,10]. The low-molecular-weight alginate with molecular weight lower than 50 kDa can be removed from the body through the kidney [11,12,13]. It is worth noting that the oxidation of alginate can strengthen its biodegradability and significantly reduce its molecular weight [14,15]. Simultaneously, the low molecular weight of oxidized sodium alginate (OSA) hydrogels is conducive to be used as the degradable hydrogel scaffolds for drug delivery system and tissue engineering for its functional groups (such as aldehyde groups) that can be quickly degraded in the body compared with natural alginate [14,16,17].

**Figure 2:**
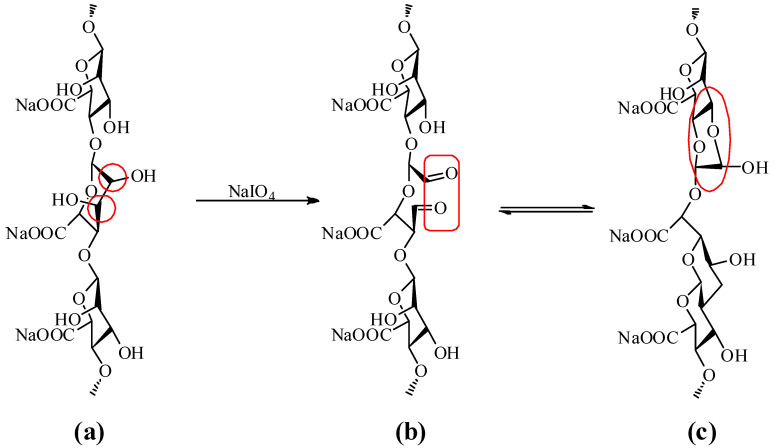
Schematic presentation of (**a**) the oxidation reaction of sodium alginate by NaIO_4_, (**b**) the formation of OSA, and (**c**) the formation of hemiacetal.

In general, the oxidation of SA can be achieved via oxidizing agents including potassium permanganate, ozone, hydrogen peroxide, and periodate. Lu et al. [18] reported that SA could be oxidized by potassium permanganate under acidic conditions, and the degradation of SA increased with the increase in the amount of potassium permanganate and the decrease in pH value of the solution. Wu et al. [19] illustrated that the free radicals produced by ozone self-decomposition could cause the degradation of SA, thereby obtaining the low molecular weight of OSA. Mao et al. [20] studied the oxidation of SA using hydrogen peroxide, which could achieve the desired molecular weight of alginate by adjusting the reaction temperature, hydrogen peroxide concentration, initial concentration of SA, and pH value in the system. To note, it can be observed that there are two adjacent -OH groups at the C-2 and C-3 positions on the repetitive unit of the alginate chain, which could be specifically oxidized by the periodate to generate the aldehyde groups [21,22]. Among the above oxidation methods, using sodium periodate (NaIO_4_) to oxidize SA is regarded as the most commonly used method to endow alginate with active functional groups and easy degradation in drug controlled delivery [23]. The oxidation of alginate with NaIO_4_ to form OSA involved C2–C3 bond breakage, thus transforming the uronic acid into an open chain adduct containing aldehyde groups. Subsequently, the generated aldehyde groups will react spontaneously with hydroxyl groups present on the adjacent uronic acid in the alginate chain to form a cyclic hemiacetal [24], as shown in Figure 2. Moreover, the carboxyl groups of alginate can be completely retained, and the oxidation degree (OD) of alginate can be also controlled by adjusting the concentration of NaIO_4_ during the oxidation process [25].

Oxidation degree (OD) is the basic parameter of OSA, which significantly affects the physical and chemical properties of OSA-based materials such as hydrogels [26], microspheres [27], and electrospinning materials [28]. The increase in OD may make OSA-based materials possess a low crosslinking degree and network density. Additionally, OD is also related to the gelling properties of alginate. Generally, alginate and its derivatives exhibit gelling ability with divalent cations [29,30,31,32] (such as Ca^2+^, Ba^2+^, Mg^2+^, Sr^2+^, etc.). However, the strength of the ionic crosslink between the OSA polymer chain and the divalent ion are weakened with the destruction of the cooperative interaction during oxidation process [23]. Fortunately, alginate with a lower OD can still form the hydrogel [25]. Therefore, it is generally necessary to select an OSA with the suitable OD before the actual application. Although some works about oxidation reactions with NaIO_4_ on the hydroxyl groups of alginate uronic units have been reported [23,24,25], the systematic characterization of the resultant OSA by multiple testing methods and the correlation between the OD on the degradability and gelation of OSA have rarely been studied at present. A large number of studies have shown a great reduction in the molecular weight of SA after being oxidized by NaIO_4_, resulting in the formation of aldehyde groups with higher reactivity [24,27,28]. The resultant OSA not only retained the water solubility, low, and biocompatibility of alginate, but also exhibited good biodegradability and better molecular flexibility. Therefore, research on the correlation between the OD of OSA on its biodegradability and gelation is beneficial to exploring the application potential of OSA-based hydrogels in the biomedical field.

Herein, to broaden the applicability of alginate, we prepared OSA with various theoretical ODs using NaIO_4_ as the oxidant. The structure and physicochemical properties of OSA was evaluated by Fourier transform infrared spectroscopy (FT-IR), ^1^H nuclear magnetic resonance (^1^H NMR), X-ray Photoelectron Spectroscopy (XPS), X-ray Diffraction (XRD), and thermogravimetric analysis (TGA). Meanwhile, gel permeation chromatography (GPC) and rheometer (DHR) were used to determine the correlation between the ODs of oxidized sodium alginate on its biodegradability and gelation. Additionally, the cytotoxicity of the formed OSA hydrogel against the MC3T3-E1 cells was also evaluated.

## 2. Materials and Methods

### 2.1. Materials

Sodium alginate (SA, M_W_ = 1,106,340, G/M = 2:1), sodium periodate (NaIO_4_), D-Glucono-δ-lactone (GDL), nanosized hydroxyapatite (HAP), and sodium dihydrogen phosphate (NaH_2_PO_4_) were bought from Aladdin Chemical Reagent Co., Ltd., Shanghai, China. Anhydrous ethanol, hydrochloric acid, potassium chloride, sodium chloride (NaCl), sodium hydroxide (NaOH), and pH 7.4 phosphate buffer solution (PBS) were obtained from Zhongyao Chemical Reagent Co., Ltd., Beijing, China. Starch and ethylene glycol were bought from Macklin Co. Ltd., Shanghai, China. All chemicals were analytical grade and were used without further purification.

### 2.2. Methods

#### 2.2.1. Synthesis of Oxidized Sodium Alginate

The oxidation of SA was carried out in aqueous solution using NaIO_4_ as the oxidant to prepare oxidized sodium alginate (OSA) with various theoretical oxidation degrees based on the reported methods [23,24]. Briefly, 5 g of SA was fully dissolved in 200 mL of distilled water in a dark bottle; then, 50 mL of anhydrous ethanol was added under vigorous stirring to obtain 2.0% (*w*/*v*) SA solution. Afterwards, a certain amount of NaIO_4_ was added to initiate the oxidation reaction at 25 °C in N_2_ atmosphere to obtain OSA. Various ratios of NaIO_4_ to the number of repetitive uronic acid of alginate (5, 10, 15, or 20 mol %) were used, as shown in Table 1. Each reaction was performed for a period of 24 h until about 10 mL of ethylene glycol was incorporated to reduce the unreacted NaIO_4_. Subsequently, 4 times the volume of anhydrous ethanol and 4 g of NaCl were added to precipitate OSA; then, they were placed in the dialysis bag with a molecular weight cutoff of 3500 to dialyze against deionized water for 5 days. Finally, the dried OSA was obtained by freeze-drying the dialyzed OSA solution.

#### 2.2.2. Determination of Oxidation Degree of OSA by UV–Vis Absorption Spectroscopy 

**Figure 3:**
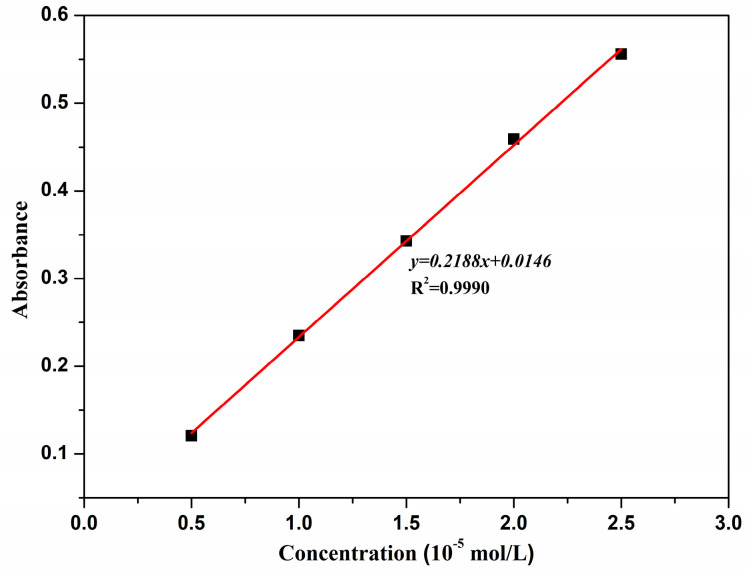
Calibration curve of NaIO_4_ as a function of its concentration.

The oxidation degree (OD) of OSA could be determined by UV–Vis absorption spectroscopy based on the difference between the initial and final amount of NaIO_4_ during the oxidation reaction. Before adding the ethylene glycol to quench the oxidation reaction, 1 mL of reaction solution was transferred and diluted to 250 mL with distilled water. Then, 3.5 mL of this diluted solution was mixed with 1.50 mL of indicator solution that was prepared by mixing equal volumes of 20% (*w*/*v*) KI and 1% (*w*/*v*) soluble starch solutions, using pH 7.0 PBS as the solvent. The absorbance of diluted mixed solution was rapidly measured with a Shimadzu UV-1800 (Shimadzu, Kyoto, Japan) UV–visible spectrophotometer at 290 nm. The concentration of NaIO_4_ in the solution was determined with a calibration curve whose linear regression equation was fitted as *y* = 0.2188 *× x*(10^−5^ mol/L) + 0.0146 (R^2^ = 0.9990), which was prepared by standard concentration of NaIO_4_ in PBS in the range of 0.5~2.5 × 10^−5^ mol/L, as presented in Figure 3. Therefore, the actual OD of OSA was calculated by the amount of NaIO_4_ according to the following equation:

(1) $$OD=\frac{N_{i}-N_{r}}{N_{o}}\times 100\%$$

where *N_i_*, *N_r_*, and *N_o_*, respectively, represent the initial moles of NaIO_4_, the residual moles of NaIO_4_, and initial moles of uronic acid of alginate.

#### 2.2.3. Characterization of Oxidized Sodium Alginate

The successful synthesis of OSA was confirmed by Fourier transform infrared spectrophotometer (FT-IR), ^1^H nuclear magnetic resonance (^1^H NMR), and X-ray Photoelectron Spectroscopy (XPS). The FT-IR measurement was performed on disks that were prepared by compressing the mixture of KBr and a slight amount of sample using a Nicolet-6700 FT-IR spectrophotometer (Thermo Scientific, Waltham, MA, USA). The FT-IR spectra of the sample were recorded in the range of wavenumbers between 4000 and 400 cm^−1^ for 64 scans with a spectral resolution of 4.0 cm^−1^. The ^1^H NMR spectroscopy of the sample that was dissolved in D_2_O (99%) with a concentration of 10 mg/mL was recorded on an AV 400 NMR nuclear magnetic resonance spectrometer (Bruker, Ettlingen, Germany) at 25 °C. The surface elemental composition of the sample was examined by XPS using an axis ultra DLD apparatus (Kratos, Manchester, UK), which was equipped with a monochromatic Al KR X-ray source operating at 15 kV, 10 mA (150 W). The spectra were collected in fixed analyzer transmission mode (FAT): survey scans at 1200~0 eV with 1.0 eV steps at an analyzer pass energy of 160 eV; narrow scans at 0.1 eV steps at an analyzer pass energy of 20 eV. The crystalline structure and thermal stability of OSA were examined by X-ray Diffraction (XRD) and thermogravimetric analysis (TGA). The XRD pattern was performed on an AXS/D8 X-ray diffractometer (Bruker, Cambridge, UK) with Cu-Kα radiation (λ = 0.154 nm). The measurement was conducted in a step scan mode at a scanning speed of 0.025°/s over a 2θ range of 5°~60°. The thermal property of OSA was determined by a Q600 thermogravimetric analyzer (TA Instrument, New Castle, DE, USA). The TGA test was carried out under a N_2_ atmosphere at a heating rate of 10 K/min with the range of 30~800 °C.

#### 2.2.4. In Vitro Biodegradation of Oxidized Sodium Alginate 

The in vitro biodegradation of OSA was performed at 37 °C using PBS buffer containing 10,000 U/mL lysozyme to simulate the physiological conditions. A total 0.2 g of OSA with various ODs was immersed in the 100-mL PBS buffer for 60 days. At different time intervals (6 days, 12 days, 20 days, 28 days, 38 days, 48 days, and 60 days), 2 mL aliquot of samples were withdrawn and their molecular weights were determined by an e2695 Gel Permeation Chromatography equipped with an Ultrahydrogel^TM^120 (7.8 × 300 mm^2^) column (Waters, Milford, MA, USA). A total 0.05% sodium azide was used as the mobile phase with a flow rate of 0.6 mL/min at 40 °C. Similarly, each time interval for the sample was tested in parallel for 3 times to take the average value. Within the time interval, the biodegradation of OSA could be evaluated by its weight-average molecular weight (M_W_).

#### 2.2.5. Gelation Ability of Oxidized Sodium Alginate 

**Scheme 1:**
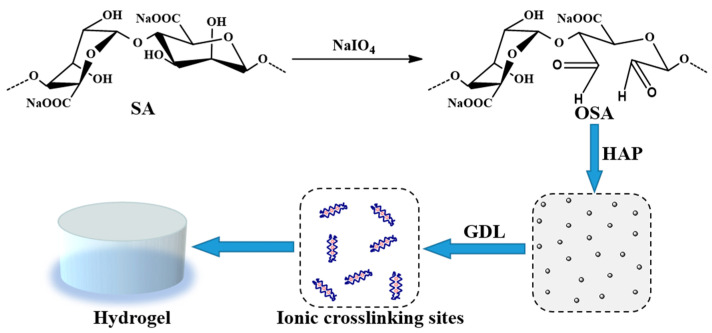
Schematic representation of the preparation of OSA and its ionic crosslinking by HAP–GDL.

To examine the gelation ability of OSA, the internal gelation of OSA was conducted based on our previous work [33]. As shown in Scheme 1, this method allowed homogeneous alginate hydrogel to be obtained by the control of the release of Ca^2+^ from the insoluble hydroxyapatite (HAP) in the presence of D-glucono-δ-lactone (GDL), which avoided the unbalanced crosslinking density, thus enhancing the mechanical property and homogeneity of the hydrogel [34,35]. In detail, a certain amount of HAP was ultrasonically dispersed in 2% (*w*/*v*) OSA solution and stirred vigorously until a homogenous solution was formed. Then, the ion cross-linking of OSA was initiated by the addition of a certain amount of GDL under the magnetic stirring. In this study, HAP–GDL complex with the molar ratio of 1:10 was used as the cross-linking system, and the molar ratio of Ca^2+^ from HAP and carboxyl from OSA was fixed at 0.18. After stirring at high speed for 3 min, the mixture was quickly transferred into a 12-well tissue culture plate and physically cross-linked at 4 °C for 24 h to obtain homogeneous OSA hydrogel. The resultant OSA hydrogel was left for 30 min to eliminate any air bubbles for further rheological measurement. The rheological properties of the OSA hydrogel with various ODs were analyzed by steady shear test and dynamic sweep measurements using a rotational rheometer with parallel-plate geometry (DHR TA Instruments, New Castle, DE, USA) at 25 °C. Steady shear measurements were carried out to record the apparent viscosity (η) with the shear rate ranging from 0.1 to 1000 s^−1^, while the oscillation frequency sweep measurements were conducted to record storage modulus G′ and loss modulus G″ with the angular frequencies (ω) ranging from 0.1 to 100 rad/s and the strain amplitude was fixed at 1%.

#### 2.2.6. In Vitro Cytotoxicity of Oxidized Sodium Alginate Hydrogel

To verify the application potential of OSA hydrogels in the biomedical field, the osteoblastic MC3T3-E1 cells cultured with the culture medium containing 90% MEM-α, 10% fetal bovine serum, 100 U/mL penicillin, and 100 μg/mL streptomycin were applied to examine their cytocompatibility by using the Cell Counting Kit-8 (CCK-8) assay. The OSA—10% hydrogels were cut into circular disks with a diameter of 20 mm and a height of 10 mm; then, they were sterilized by cobalt 60 radiation with the irradiation intensity of 8 kGy. The MC3T3-E1 cells were seeded on the OSA—10% hydrogels in the 24-well tissue culture plates at a density of 5 × 10^4^ per well, while the same cells were seeded on the tissue culture plates as a blank control. Afterwards, the culture medium was replenished to make the total amount of medium per well reach up to 500 μL. Subsequently, they were transferred to an incubator containing 5% CO_2_, 95% air, and 100% relative humidity at 37 °C and their culture media were replaced every 2 days. After 2 days and 5 days incubation, 50 μL of CCK-8 reagent was added to 500 μL of medium in each well, and they were placed in the incubator at 37 °C for 4 h. Finally, 100 μL of solution from each well was transferred to a 96-well plate, whose absorbance value (OD) was determined by an X-mark microplate reader (Bio-rad, Hercules, CA, USA) at a wavelength of 450 nm. Since the OD of the cell medium on the OSA—10% hydrogel is proportional to the number of living cells, the cell viability of the MC3T3-E1 cells on the OSA—10% hydrogel could be judged by comparing the OD values.

## 3. Results and Discussion

### 3.1. Oxidation of Alginate via NaIO_4_

Since there are a large number of hydrophilic groups such as hydroxyl and carboxyl groups on the backbone of alginate molecules, they can easily form intramolecular hydrogen bonds, resulting in the strong and rigid molecular structure of alginate, which may restrict its scope of application in drug delivery [36,37]. However, the inert dihydroxy groups on the alginate backbone can be oxidized to generate the reactive dialdehyde groups by exploiting the oxidative properties of NaIO_4_, which significantly improved its biodegradability [23,38]. The cleavage of the C2–C3 bond of alginate uronic acid monomer could occur in the oxidation reaction of NaIO_4_, which strictly destroyed the rigid structure of the alginate molecular backbone, thus enhancing its molecular flexibility [24]. In this work, the SA was partially oxidized to a theoretical extent (5%, 10%, 15%, and 20%) and the oxidation degree (OD)—defined as the percentage of oxidized uronic acid groups in the alginate—was determined by the consumption of NaIO_4_ with the results shown in Table 1. It was obvious that the OD of OSA increased with the increase in the amount of NaIO_4_, and the actual OD was close to the theoretical OD. This result directly indicated that most of the added NaIO_4_ participated in the oxidation reaction, and the oxidation reaction of alginate with NaIO_4_ was feasible and active.

### 3.2. Molecular Structure and Thermal Stability of OSA

**Figure 4:**
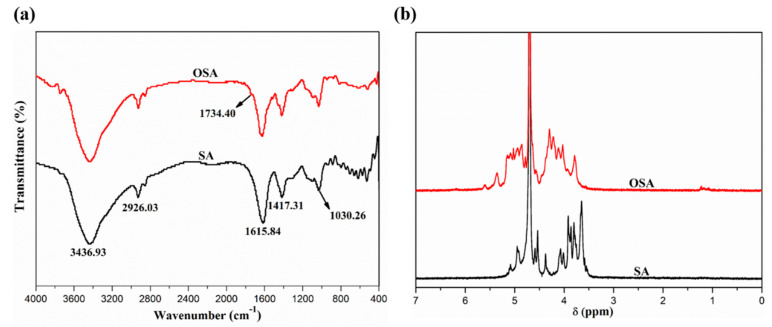
(**a**) FT-IR spectra and (**b**) ^1^H NMR spectra of SA and OSA.

The molecular structure and specific functional groups of the resultant OSA could be confirmed by FT-IR and ^1^H NMR spectroscopy. As shown in Figure 4a, both SA and OSA revealed the broad hydroxyl stretching vibration absorption peaks in the range of 4000~3000 cm^−1^ [39]. In detail, SA exhibited main characteristic peaks at 2926, 1616, and 1417 cm^−1^ owing to the C-H stretching vibration of the polysaccharide structure and the asymmetric and symmetric stretching vibration of -COO^−^ [40]. Additionally, the absorption peaks at 1030 cm^−1^ were attributed to the C-O stretching vibration on the polysaccharide skeleton [41]. In comparison with SA, OSA had a new characteristic peak at 1734 cm^−1^, which was assigned to the vibration absorption peak of the C=O bond on the aldehyde group. This peak was too weak to be detected for the hemiacetal formation of the free aldehyde groups [42]. In addition, the hydroxyl stretching vibration absorption peak at 3437 cm^−1^ in the spectrum of SA became blue-shifted to 3443 cm^−1^ in the spectrum of OSA, implying a decline in the amount of -OH groups of alginate. These results indicated that the adjacent hydroxyl groups at the C-2 and C-3 positions on alginate uronic acid monomer were oxidized to aldehyde groups by NaIO_4_ [25,41]. Moreover, OSA displayed similar FT-IR spectroscopy results to the raw SA, which demonstrated the periodate ion only cleaved the C2–C3 linkage by the oxidation reaction, leading to the formation of a dialdehyde [24].

Figure 4b shows the ^1^H NMR spectra of SA and OSA. The proton peaks of SA and OSA ranging from 5.0 to 3.5 ppm were assigned to the hydrogen atoms of native alginate backbone [40]. Compared with SA, the two new proton peaks were discovered at 5.3 and 5.6 ppm in the spectrum of OSA, which were attributed to a hemiacetalic proton generated from the hydroxyl groups of aldehyde and its neighbors, confirming the achievement of the oxidation with NaIO_4_ [23]. Moreover, the proton peaks of SA appearing at 3.7~3.55 ppm that were assigned to the H2 of α-l-guluronic acid decreased and moved to the high field for cleavage of the C2–C3 bond of the uronic acid monomer [24]. The appearance of these new proton signal peaks and the change of the proton signal peaks also directly proved the successful oxidation of alginate with NaIO_4_.

**Figure 5:**
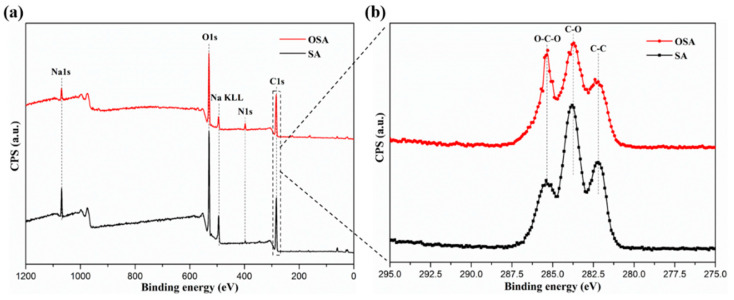
(**a**) XPS spectra and (**b**) XPS C1s narrow scans of SA and OSA.

As a complementary technique, XPS was performed to further verify the surface composition of resultant OSA. As shown in Figure 5a, XPS spectra of SA and OSA confirmed the presence of the anticipated peaks for Na (1069.02 eV), O (530.23 eV), N (397.54 eV), and C (284.68 eV), with an O/Na ratio close to the theoretical value of 6 [43], indicating that partial oxidation of alginate by NaIO_4_ preserved the main structure and functional groups of alginate. In addition, the peak assignment of XPS C1s narrow scans of SA and OSA that could elucidate the bonding atmosphere has been marked in the spectra in Figure 5b according to the previous report [44]. It can be observed that the C1s narrow scan revealed three peaks at 285.46 eV (O–C–O), 283.87 eV (C–O), and 282.20 eV (C–C), respectively. It is found that the peak ratio of the O–C–O species to the C–O species in the C1s narrow scan of OSA was significantly higher than that of SA, due to the partial oxidation of alginate by NaIO4, which was consistent with previous results reported by Jejurikar et al. [24]. Thus, these results further verified the successful partial oxidation of alginate by NaIO_4_.

**Figure 6:**
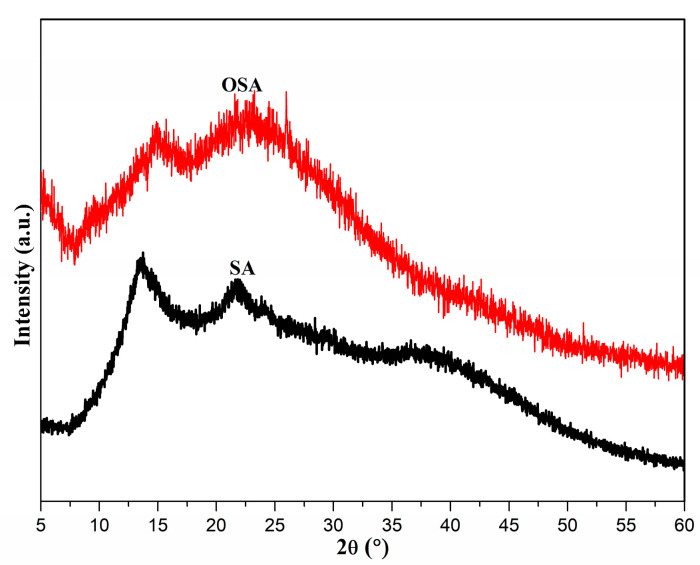
XRD patterns of SA and OSA.

The crystal structure of OSA was evaluated by XRD. As shown in Figure 6, the XRD patterns of SA and OSA were proved to be amorphous structures, consistent with previous reports [45]. The typical characteristic diffraction peaks of SA at 2θ = 14.5° and 22.0° represented the hydrated crystalline structure generated from the intramolecular hydrogen bonds of SA [46]. Compared with SA, the diffraction peaks of OSA were shifted to 2 θ = 14.9° and 23.0°, and the peak at 2 θ = 23.0° was a broader peak. These results implied that the structure of SA was changed during the oxidation process and the intermolecular hydrogen bonds were weakened with the decrease in molecular weight.

**Figure 7:**
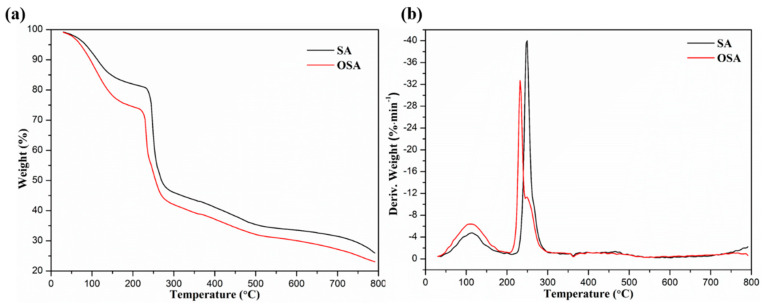
(**a**) TGA curves and (**b**) DTG curves of SA and OSA.

Thermogravimetric analysis was the most effective method to characterize the different thermal stability of SA and OSA, which could indirectly reflect the change of molecular structures of alginate during the oxidation reaction. During the test, since the mass of the samples will change with the increase in temperature, the thermal stability of SA and OSA can be judged by comparing the initiating decomposition temperature and the final weight loss rate [47]. As shown in Figure 7a, the weight loss of the samples at the temperature below 100 °C corresponds to the evaporation of water in the samples. Major weight loss occurred at the temperature ranging from 200 °C to 300 °C, which resulted from dehydration of hydroxyl group along the alginate backbone and its thermal decomposition of hexuronic segments [48,49]. The total weight loss rates of SA and OSA at 800 °C were 74% and 77%, respectively. Compared with SA, OSA possessed lower residual weight, which may be ascribed to the oxidation of hydroxyl groups of SA. From the DTG curves of SA and OSA in Figure 7b, it can be observed that both SA and OSA displayed two main weight loss stages. The maximum rate of the first weight loss stage occurred at 100~150 °C, which was ascribed to the removal of physically adsorbed water and bound water from the polymer materials [27,28], while the second weight loss stage occurred at 200~300 °C and was attributed to the thermal decomposition of the polymer materials, which was gradually thermally cracked into CO, CO_2_, and H_2_O, resulting in a rapid decline in their weight [33]. The temperatures corresponding to the maximum rates of the thermal decomposition weight loss of SA and OSA were 249.6 and 232.2 °C, respectively. Obviously, the initiating thermal decomposition temperature of OSA was lower than that of raw SA, indicating that the thermal stability of OSA decreased after the oxidation reaction. This result was ascribed to the destruction of the intramolecular hydrogen bond for the cleavage of the C2–C3 bond of alginate uronic acid monomer during the oxidation process, thereby improving the molecular flexibility of alginate.

### 3.3. In Vitro Biodegradation Analysis

**Figure 8:**
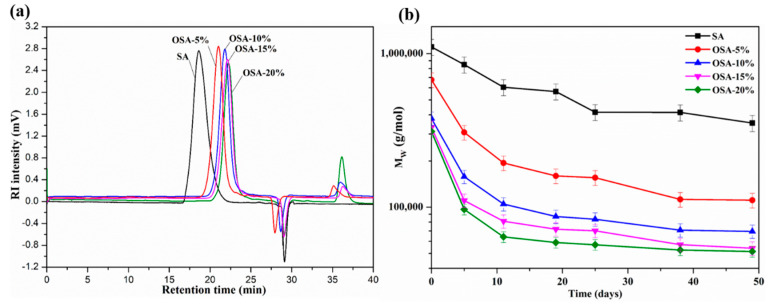
(**a**) GPC chromatographs of SA and OSA with various OD; (**b**) biodegradation curves of SA and OSA with various OD in PBS solution.

The weight-average molecular weights (M_W_) of the water-soluble OSA were measured via GPC, which used 0.05% sodium azide as the mobile phase. Figure 8a depicts GPC chromatographs of SA and OSA with various ODs. It can be observed that the molecular weight of OSA decreased with an increase in the OD of OSA, similar to the previous results [50]. The SA and OSA with various ODs were immersed in the PBS buffer at 37 °C for in vitro biodegradation, and the biodegradation performance of the samples was studied by measuring their molecular weight at a specific time. As shown in Figure 8b, SA underwent less biodegradation because SA was hardly biodegradable in the body [9,10]. In contrast, OSA showed significant biodegradability, especially within 10 days of the onset of biodegradation, which could exhibit great potentials as the degradable hydrogel scaffolds or controlled release drug delivery system in biomedical field. Additionally, the M_W_ of OSA decreased very rapidly with the increase in their OD, and all of the OSA with various ODs revealed similar biodegradation trends. These behaviors were attributed to the cleavage of the C2–C3 bond of alginate uronic acid, as oxidation occurred because this was a free-radical-independent biodegradation process, which showed that the biodegradation depended directly on the concentration of NaIO_4_ in the reaction solution [23]. Consequently, the biodegradation rate of alginate could be controlled by regulating the OD of OSA to meet different application requirements.

### 3.4. Gelation Study of OSA

**Figure 9:**
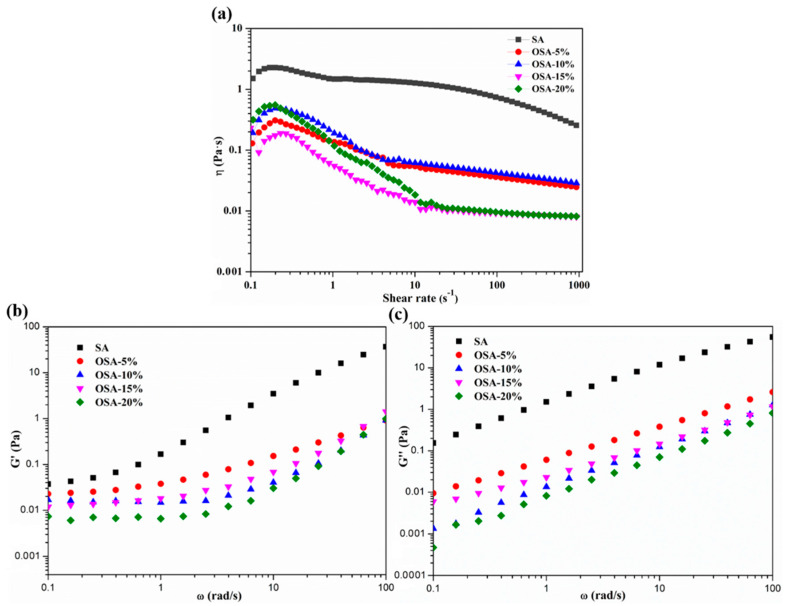
(**a**) Viscosity of SA hydrogel and OSA hydrogel with various ODs as a function of shear rate at 25 °C; (**b**) viscosity of SA hydrogel and OSA hydrogel with various ODs as a function of shear rate; (**c**) storage (G′) and loss (G″) moduli of SA hydrogel and OSA hydrogel with various ODs as functions of angular frequency (ω).

OSA with oxidation degrees ranging from 5% to 20% was formulated into the corresponding concentration of homogeneous hydrogel, and their gelation ability was preliminarily determined by observing the results of the viscosity variation with the shear rate, as shown in Figure 9a. Since the formation time of hydrogel was related to the molecular weight of the oxidized alginate derivatives, high molecular weight of OSA could greatly reduce the formation time of hydrogel. It could be found that the viscosity (η) of the SA hydrogel exhibited a slight variation with the shear rate, which displayed typical Newtonian liquid characteristics. This result was mainly attributed to the highly stretched rigid molecular structure of SA [37]. On the contrary, the OSA hydrogel with various ODs displayed apparent shear-thinning behavior, which was ascribed to the destruction of the intramolecular hydrogen bond for cleavage of the C2–C3 bond of the alginate uronic acid monomer during the oxidation process. In comparison with SA hydrogel, the OSA hydrogel exhibited distinctly lower viscosity (η) at the same shear rate, indicating that the gelation ability of OSA significantly decreased due to the oxidation of the adjacent hydroxyl groups at C-2 and C-3 positions on alginate uronic acid monomer [24]. Simultaneously, the viscosity of OSA hydrogel decreased with the increase in OD at the same shear rate, which indicated that the hydrogel could be prepared by controlling the OD. 

**Figure 10:**
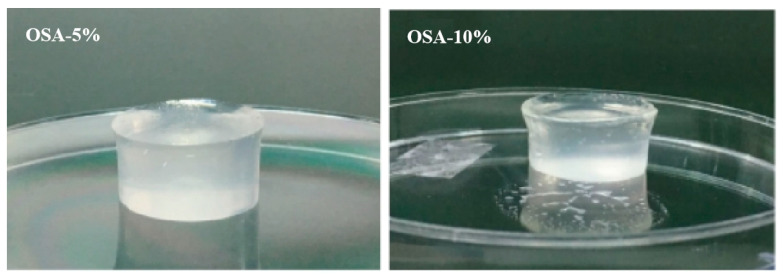
The physical picture of OSA—5% and OSA—10% hydrogel.

In addition, it can be also observed from Figure 9b,c that the SA hydrogel and OSA hydrogel with various ODs presented an upward trend for their storage modulus (G′) and loss modulus (G″) as the angular frequency (ω) increased. In general, the storage modulus (G′) and loss modulus (G″) were, respectively, related to the strength of the internal structure and dynamic viscosity. By contrast, the SA hydrogel exhibited relatively better mechanical properties as the storage (G′) and loss (G″) moduli of SA hydrogel were apparently higher than that of OSA hydrogel at the same angular frequency (ω). Furthermore, a reduction in both of the storage (G′) and loss (G″) moduli was found when the OD increased, which could be owing to the decrease of the molecular weight. Thus, it could be concluded that the oxidation reaction would cause the decrease in the molecular weight of the alginate, further affecting the formation of hydrogel. As presented in Figure 10, only OSA with ODs of 5% and 10% could be cross-linked by Ca^2+^ to form hydrogel, while no hydrogels were formed when the OD exceeded 10%. Therefore, the critical OD of OSA to form a hydrogel was 10%, which was consistent with the previous report [23]. Meanwhile, OSA with low molecular weight was beneficial to biomedical applications, such as regenerative medicine, 3D-printed/composite scaffolds, and tissue engineering, because it was found that, for M_W_ ≤ 50 kDa, alginate could be removed from the human body [11,12].

### 3.5. Cytocompatibility of OSA Hydrogel

**Figure 11:**
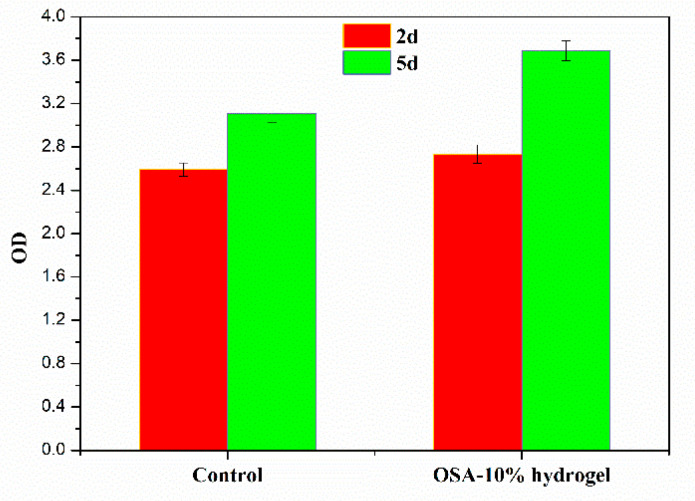
Cell viability of MC3T3-E1 cells cultured on the OSA—10% hydrogel for 2 and 5 days, respectively.

After 2 days and 5 days incubation, the in vitro cytotoxicity of OSA—10% hydrogel was measured using the CCK—8 assay kit. As shown in Figure 11, the MC3T3-E1 cells displayed better proliferative activity on the OSA—10% hydrogel than that on the control group, indicating that the MC3T3-E1 cells could survive on the OSA—10% hydrogel and grow in their 3D pore structure. These results further indicated that the OSA hydrogel had no cytotoxicity, presenting excellent biocompatibility. Therefore, the OSA hydrogel with excellent cytocompatibility could realize their extended biomedical applications in regenerative medicine, 3D-printed/composite scaffolds, and tissue engineering.

## 4. Conclusions

In this work, the oxidation of SA with various ODs was achieved using NaIO_4_ as the oxidant, and the structure and properties of the resultant OSA were characterized by multiple testing methods. Meanwhile, the effect of OD on the biodegradability and gelation ability of OSA was also investigated. The adjacent hydroxyl groups at C-2 and C-3 position on alginate uronic acid monomer were oxidized to aldehyde groups by NaIO_4_. On account of the cleavage of the C2–C3 bond of alginate uronic acid monomer during the oxidation process, the thermal stability of OSA was worse than that of SA. On the contrary, OSA possessed smaller molecular weight and better degradability in contrast to SA; the higher the OD of OSA, the better the degradability. Nevertheless, the gelation ability of OSA decreased with the increase in OD due to the decrease in molecular weight. It is worth noting that the critical OD of OSA to form a hydrogel was 10%. Thus, it could be concluded that both the formation and biodegradability of OSA hydrogel were controlled by the OD. This work aimed to improve the properties of alginate via the oxidation reaction to broaden its application in the biomedical field.

## Figures and Tables

**Table 1:** Oxidation reaction parameters with NaIO_4_ for OSA with various OD.

Theoretical OD	Absorbance	Mole of Alginate Uronic Acid (mmol)	NaIO_4_ (g)	Actual OD
5%	0.002	1.2625	0.27	4.99%
10%	0.020	2.5250	0.54	9.90%
15%	0.016	3.7875	0.81	14.91%
20%	0.018	5.0500	1.08	19.88%

## Data Availability

The data presented in this study are available on request from the corresponding author. Samples of the compounds are available from the authors.
